# Platelet-rich plasma does not reduce skeletal muscle fibrosis after distraction osteogenesis

**DOI:** 10.1186/s40634-018-0143-7

**Published:** 2018-07-13

**Authors:** Ichiro Tonogai, Fumio Hayashi, Toshiyuki Iwame, Tomoya Takasago, Tetsuya Matsuura, Koichi Sairyo

**Affiliations:** Department of Orthopedics, Institute of Health Biosciences, Tokushima University Graduate School, 3-18-15 Kuramoto, Tokushima, 770-8503 Japan

**Keywords:** Platelet-rich plasma, Distraction osteogenesis, Skeletal muscle, Fibrosis

## Abstract

**Background:**

Skeletal muscle fibrosis caused by an increase in collagen deposition often occurs after distraction osteogenesis. Although studies are available reporting the effects of platelet-rich plasma (PRP) on tissue healing following injury, current findings remain controversial. This study focused on determining whether PRP reduces skeletal muscle fibrosis caused by distraction osteogenesis.

**Methods:**

Tibial osteotomies were performed on 8-week-old wild type mice, and tibiae were distracted at a rate of 0.42 mm/day for 2 weeks, starting 1 week after osteotomy. Immediately after distraction was completed (3 weeks after osteotomy), PRP or phosphate buffered saline (as a sham) was injected into the gastrocnemius (GC) muscle. The GC muscles were harvested and analyzed.

**Results:**

The amount and area of collagenous tissue increased in both the PRP and control groups following distraction osteogenesis, but the changes were not significantly different between both groups at all time points (*p* = 0.89, 0.45, 0.33 and 0.52 at 4, 6, 8 and 10 weeks).

**Conclusion:**

From this study, our results suggest that PRP did not significantly reduce skeletal muscle fibrosis due to distraction osteogenesis.

## Background

Distraction osteogenesis is a useful treatment for trauma and musculoskeletal disorders that result in limb length discrepancy and short stature (Ilizarov 1990). However, distraction osteogenesis causes a specific kind of skeletal muscle damage (Williams et al. 1999; Williams et al. 1998; Williams et al. 2001; De Deyne et al. 2000; Antoci et al. 2006; Tonogai et al. 2015). This extremely severe skeletal muscle damage is characterized by repeated regular and gradual distraction of the muscle during distraction phase (Tonogai et al. 2015). Fibroblasts migrate to the damaged site and proliferate, and fibrous tissue is formed by increased production of collagen (Tonogai et al. 2015). Therefore, fibrosis often results in common clinical complications such as skeletal muscle contracture (Antoci et al. 2006; Paley 1999).

Platelet-rich plasma (PRP), an easily obtainable product, is the plasma fraction derived from a person’s own blood, and it contains high concentrations of platelets including growth factors (GFs), which are known to play a critical role in tissue healing (Molloy et al. 2003; Anitua et al. 2006; Wang et al. 2006; DeLong et al. 2012). However, not all studies have found positive outcomes of PRP (Nin et al. 2009; de Vos et al. 2010; de Jonge et al. 2011; Schepull et al. 2011). In terms of skeletal muscle, some authors report that PRP enhances the skeletal muscle healing process (Menetrey et al. 2000; Huard et al. 2002; Wright-Carpenter et al. 2004; Järvinen et al. 2007; Sanchez et al. 2007; Cunha et al. 2014; Contreras-Muñoz et al. 2017), while Harris et al. report the development of thrombosis, necrosis, and calcium deposition histologically at intramuscular PRP injection sites and thus the potential for negative effects of PRP injections (Harris et al. 2012), and Kelc et al. report concerns that PRP injection may lead to fibrosis (Kelc et al. 2015).

Some articles describe the osteogenic effect of PRP injection during osteogenesis (Kitoh et al. 2004; Ali et al. 2015). However, no studies have investigated the effect of PRP on skeletal muscle fibrosis caused by distraction osteogenesis, although we reported the evaluation of the skeletal muscle after distraction osteogenesis in mice without any additional intervention such as PRP administration (Tonogai et al. 2015). We hypothesized that PRP has favorable effects on skeletal muscle fibrosis following distraction osteogenesis, and in this study we investigated specifically whether PRP reduces skeletal muscle fibrosis and what ratio PRP influences on skeletal muscle fibrosis following distraction osteogenesis in mice.

## Methods

### Experimental animals

Eight-week-old wild-type (WT) male (C57BL/6xDBA/2) F1 (BDF1) mice (weight 23–27 g) purchased from Japan SLC Inc. (Shizuoka, Japan), were used in this study. All animal experiments were approved by the Animal Care and Use Committee of Tokushima University Graduate School and were performed according to the Guidelines for the Care and Use of Laboratory Animals at Tokushima University Graduate School.

### Distraction osteogenesis

The operative procedure was performed with an external fixator for limb lengthening as described previously (Tonogai et al. 2015). The external fixator consisted of 2 open aluminum rings, 2 stainless steel screws, and 6 nuts. Mice were anesthetized with an intraperitoneal injection of 2.5% tribromoethanol. The external fixator was applied with super glue to the right tibia, using two 27 G needles for each proximal and distal ring. A transverse osteotomy was performed with a No. 11 scalpel blade at the mid-shaft of the tibia between the 2 rings without any additional soft tissue damage. The wound was closed with 5–0 nylon suture after completion of the osteotomy. The mice were allowed free unrestricted weightbearing.

**Fig. 1:**
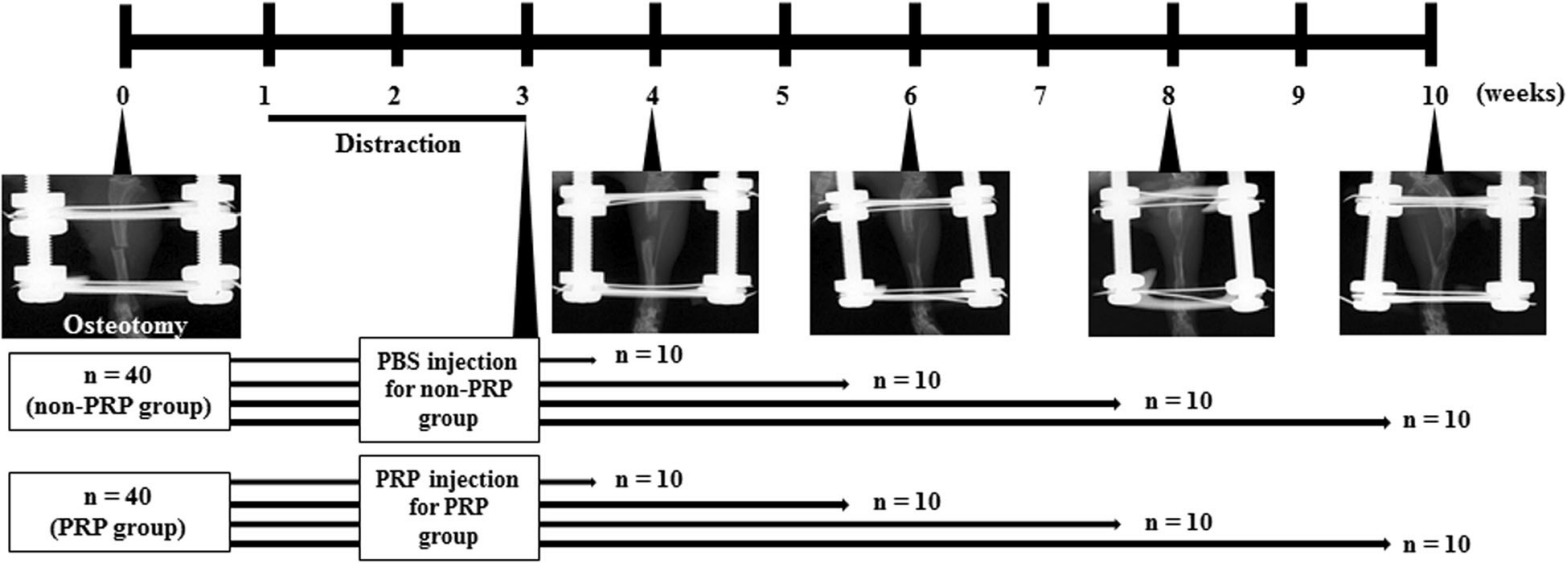
Experimental protocol and representative radiographs of distraction osteogenesis from 0 to 10 weeks after osteotomy

The protocol of the distraction osteogenesis is shown in Fig. 1. We performed distraction osteogenesis of the right hind limbs for total 80 mice, and assigned 40 mice into non-PRP group and the remaining 40 mice into PRP group, and 10 mice in each group were sacrificed at 4, 6, 8, and 10 weeks after osteotomy (*n* = 10/group/week). Briefly, after a lag phase of 1 week, distraction was carried out at a rate of 0.21 mm every 12 h for 2 weeks, because a faster rate might cause peroneal nerve palsy resulting in denervation change of the muscle and a slower rate might lead to less muscle fibrosis. The length of the distraction gap seen on X-ray was approximately 6 mm at the end of the distraction phase. PRP 40 μl was injected directly into each distracted right gastrocnemius (GC) muscle in a total of 40 mice (PRP group) immediately after completing distraction osteogenesis (3 weeks after osteotomy), because muscle fibrosis was very severe at that time point as we reported previously (Tonogai et al. 2015). The injection site was prepped in a typical sterile fashion, and the needle was exactly inserted through the skin into the belly of the right GC muscle under ultrasound. Half of the volume, 20 μl, was injected into the medial right GC, and the remaining half was injected into the lateral right GC. For the sham group, 40 μl phosphate buffered saline (PBS) was injected into each right distracted GC muscle in a total of 40 mice in the same manner (non-PRP group).

### PRP preparation

**Fig. 2:**
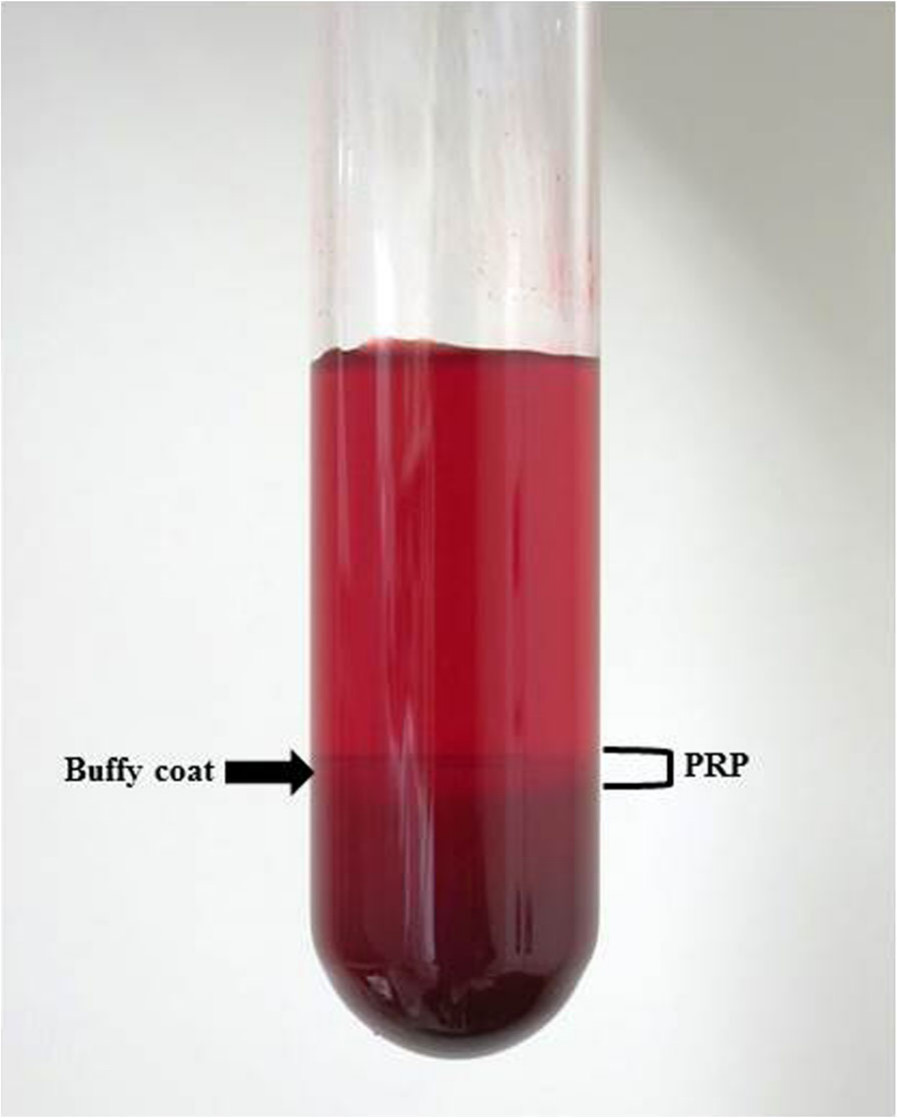
Whole blood in a tube containing ACD, which was anticoagulant, after centrifuging shows 3 layers comprising clear plasma, buffy coat including PRP component, and RBC layer

We prepared allogenic PRP-containing leukocytes (L-PRP) using the BS Medical Platelet Concentration System (BS Medical, Tokyo, Japan), which was specialized for small animals like mouse, following the manufacturer’s instructions. L-PRP were prepared in a two-step centrifugation process. Briefly, 500 μl was withdrawn via intra-cardiac puncture from 1 adult male mouse (11 weeks old). Then, 5 ml whole blood from 10 mice was pooled and collected in a blood tube including 0.5 ml acid citrate dextrose (ACD) solution which was anticoagulant. The whole blood was centrifuged at 550 G (1800 rpm) for 8 min, resulting in the formation of 3 layers: plasma, buffy coat including PRP component, and red blood cell (RBC) layer (Fig. 2). As much as possible, the top two-thirds of the supernatant was obtained for PRP preparation and was centrifuged again at 380 G (1500 rpm) for 6 min, resulting in the formation of 3 layers: plasma, buffy coat including PRP component, and a small RBC layer. The buffy coat including 500 μl PRP was carefully isolated and harvested from the tube. In this study, a total of 20 ml of whole blood was collected from 40 adult male 11-week-old mice, and a total of 2 ml PRP was obtained. PRP was used immediately after preparation rather than storing for later use, to minimize any effects of prolonged storage or freeze/thaw cycles and in accordance with how it is typically prepared and applied clinically (Delos et al. 2014). Next, 40 μl of PRP was injected into each GC of the distracted hind limb (20 μl into the medial GC and 20 μl into the lateral GC) in a total of 40 mice (PRP group) just after distraction osteogenesis finished. The volume of injection was consistent with another study using a mouse muscle injury model (Qu and Huard 2000; Ota et al. 2011; Terada et al. 2013). The remaining 400 μl PRP was subjected to measurement, consisting of white blood cell (WBC), RBC, and platelet counts, and assessment of the GFs vascular endothelial growth factor (VEGF), platelet-derived growth factor-AB (PDGF-AB), and transforming growth factor–β1 (TGF-β1).

### Assessment of WBC, RBC, platelets, and GFs

WBC, RBC, and platelet counts in whole blood and PRP were determined using an automated cell counter to determine whether platelets were enriched in PRP. Concentrations of VEGF (R&D Systems, Minneapolis, MN, US), PDGF-AB (R&D Systems, Minneapolis, MN, US), and TGF-β1 (R&D Systems, Minneapolis, MN, US) were determined by enzyme-linked immunosorbent assay (ELISA) to detect and quantify the presence of GFs. Analyses were then performed according to the manufacturer’s instructions. Briefly, samples and standards were added to 96 GF antibody-coated well plates. After incubation and removal of unbound substances, a secondary antibody was added. This was followed by further washing and incubation with a substrate. The color reaction was stopped, and optical density was measured at a wavelength of 495 nm.

### Tissue preparation

We chose GC muscle because we could obtain enough muscle volume to evaluate in histological analysis and hydroxyproline assessment, although tibialis antetior (TA) muscle also underwent fibrosis. Right GC muscles in each group were harvested, weighed, and flash frozen in liquid nitrogen-precooled isopentane at 4, 6, 8, and 10 weeks after osteotomy (*n* = 10/group/week). Then, all samples were cut longitudinally in the middle of the right GC muscle and divided into medial and lateral parts. The medial GC muscles from each group per week were used for histological analysis (*n* = 10/group/week). The lateral GC muscles from each group per week were used for hydroxyproline assay to assess collagen content (*n* = 10/group/week).

### Histological analysis

A cryostat was used to make 10-μm thick transverse sections of the central part of the belly of the GC muscle. Sections were mounted on MAS-coated slides (Matsunami Glass Industries, Osaka, Japan) and fixed in ice-cold acetone for 10 min. Sections were stained with Masson’s trichrome for histological examination of skeletal muscle fibrosis. The degree of fibrosis was evaluated in Mason’s trichrome staining images. Fibrotic blue area was measured in all fields using the image analysis software WinROOF (Mitani Corporation, Fukui, Japan), and was calculated to estimate the percentage of fibrosis development.

### Hydroxyproline assay for collagen content

Hydroxyproline is a major constituent of collagen and is highly expressed in fibrous tissue (Mu et al. 2010). Total hydroxyproline content was determined in the GC muscle using a colorimetric assay kit (BioVision, Milpitas, CA) to evaluate fibrotic changes in the distracted GC muscles, as described previously (Tonogai et al. 2015). Briefly, GC muscles were hydrolyzed in 12 N HCl at 120 °C for 3 h. After hydrolysis, samples were neutralized and treated with chloramine T solution for 5 min at room temperature followed by dimethylamine borane solution for 90 min at 60 °C. Absorbance was measured at 560 nm. Hydroxyproline concentration was determined by comparison with a standard curve prepared from pure hydroxyproline of known concentrations.

### Statistical analysis

Data are expressed as mean ± standard error of mean (SEM). Unpaired Student’s t-test was used to analyze differences between the PRP and non-PRP groups at 4, 6, 8, and 10 weeks after osteotomy. *P* < .05 was considered statistically significant.

## Results

### Hematological analysis of WBC, RBC, and platelets

The WBC count was approximately 2.5 times higher in PRP (11,200/μl) than in whole blood (4500/μl), indicating that the prepared PRP was actually L-PRP (Table 1). The RBC count was very low in PRP (4 × 10^4^/μl) compared with whole blood (757 × 10^4^/μl; Table 1). The platelet count was approximately 7.7 times higher in PRP (162 × 10^4^/μl) than in whole blood (21 × 10^4^/μl) (Table 1).

**Table 1:** WBC, RBC, and platelet counts in whole blood and PRP

	WBC (/μl)	RBC (×10^4^/μl)	Platelet (×10^4^/μl)
Whole blood	4500	757	21
PRP	11,200	9	162

Normal values: WBC 5000–13,700/μl, RBC 790–1010 × 10^4^/μl, Platelet 60–120 × 10^4^/μl

*WBC* white blood cell, *RBC* red blood cell, *PRP* platelet-rich plasma

### Measurement of GFs using ELISAs

The concentration of VEGF, PDGF-AB, and TGF-β1 were all higher in PRP than in plasma (Table 2). VEGF was approximately 1.7 times higher at 69.7 pg/ml; PDGF-AB was approximately 3.4 times higher at 9.8 ng/ml; and TGF-β1 was approximately 7.4 higher at 700.2 ng/ml.

**Table 2:** Concentration of VEGF, PDGF-AB, and TGF-β1 in plasma and PRP

	VEGF (pg/ml)	PDGF-AB (ng/ml)	TGF-β1 (ng/ml)
Plasma	41.6	2.9	94.0
PRP	69.7	9.8	700.2

*VEGF* vascular endothelial growth factor, *PDGF-AB* platelet-derived growth factor-AB, *TGF-β1* transforming growth factor-β1, *PRP* platelet-rich plasma

### Muscle wet weight

**Fig. 3:**
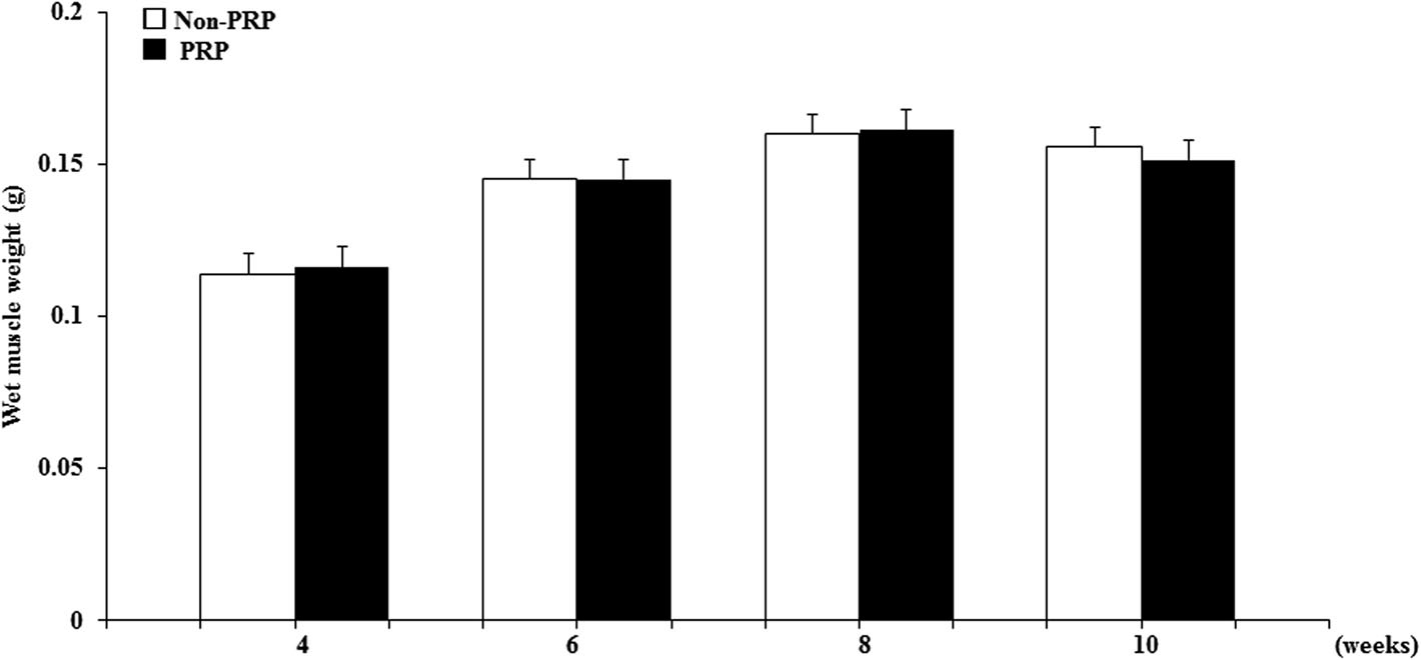
Time course of changes in wet weight of GC muscles of the non-PRP and PRP groups at 4, 6, 8, and 10 weeks after osteotomy. Data represent mean ± SEM (*n* = 10/group/week)

We evaluated muscle wet weight, because it included moisture reflecting muscle edema. Wet weight of the GC muscles in the non-PRP and PRP groups decreased at 4 weeks after osteotomy (0.114 ± 0.002 g and 0.116 ± 0.004 g, respectively) and then gradually increased from 6 weeks (Fig. 3). Muscle weight improved to 0.160 ± 0.009 g in the non-PRP group and 0.161 ± 0.006 g in PRP group at 8 weeks, and to 0.155 ± 0.005 g and 0.151 ± 0.007 g, respectively, at 10 weeks. Thus, findings at 8 and 10 weeks were almost the same as the 0-week level in both groups (Fig. 3). Weight in the PRP group did not differ significantly from that of the non-PRP group at any time point (*p* = 0.68, 0.91, 0.89 and 0.46 at 4, 6, 8 and 10 weeks).

### Fibrosis

**Fig. 4:**
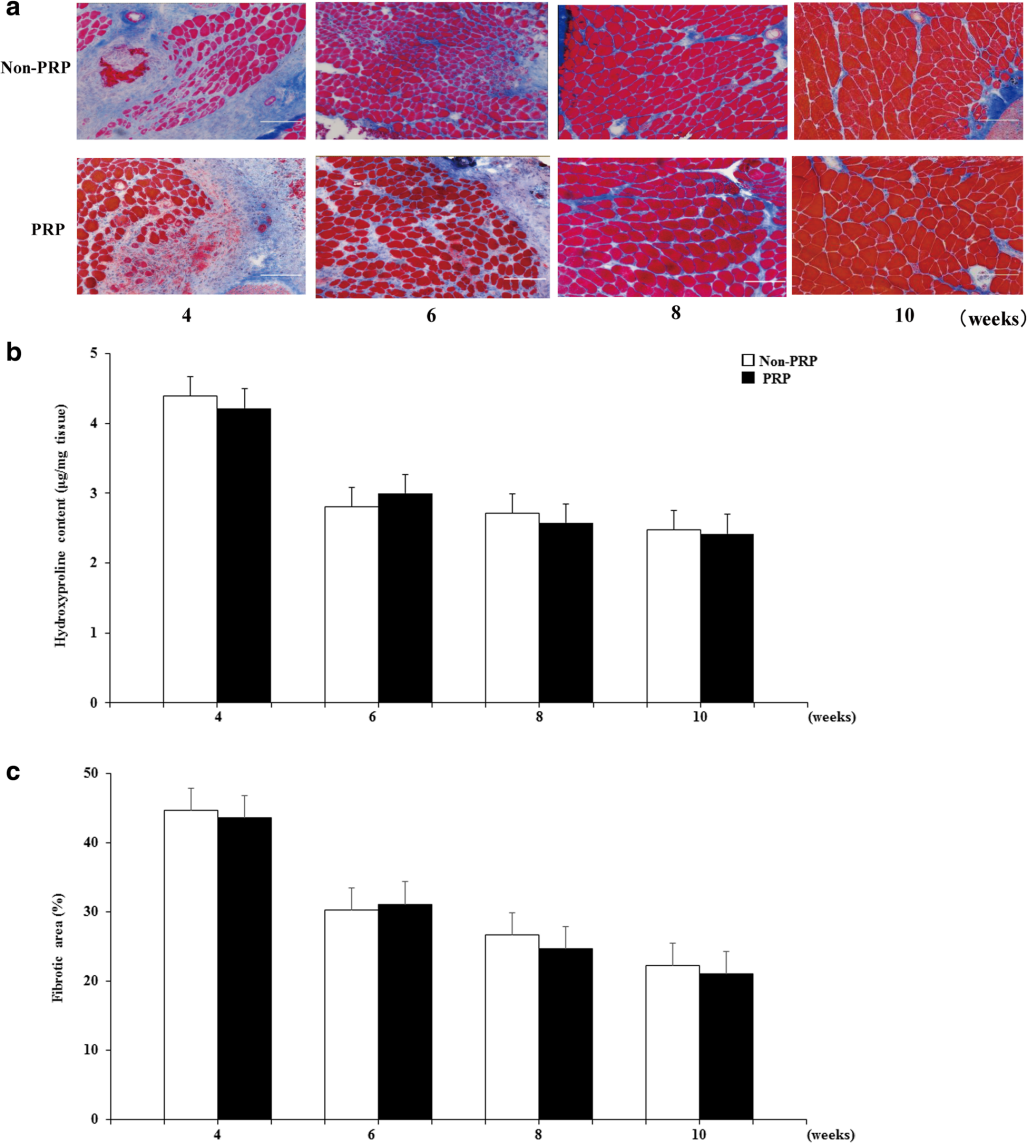
**a** Representative cross-sections of GC muscles stained with Masson’s trichrome in the non-PRP and PRP groups at 4, 6, 8, and 10 weeks after osteotomy (Bar, 100 μm). **b** The ratio of the fibrotic area to the total muscle cross sectional area in the GC muscles of the two groups. Data represent mean ± SEM (*n* = 10/group/week). **c** Total hydroxyproline content in the GC muscles of the two groups. Data represent mean ± SEM (*n* = 10/group/week)

Masson’s trichrome staining of representative images of non-PRP and groups PRP are shown in Fig. 4a. The appearance of severely fibrotic areas was similar in both groups at 4 weeks after osteotomy. The area of collagen deposition gradually decreased but persisted in both groups at 6, 8, and 10 weeks, at similar levels in both groups. The ratio of the fibrotic area to the total cross-sectional area was of the GC muscles in both groups remarkably increased at 4 weeks after osteotomy (non-PRP: 44.6 ± 1.5%; PRP: 43.7 ± 1.5%) and then gradually decreased at 6, 8, and 10 weeks (Fig. 4b). At 6, 8, and 10 weeks, there were no significant differences between the non-PRP and PRP groups (30.3 ± 0.6% vs 31.1 ± 1.4%, *p* = 0.73; 26.6 ± 1.1% vs 24.6 ± 1.2%, *p* = 0.35; and 22.3 ± 1.0% vs 21.1 ± 0.8%, *p* = 0.49, respectively; Fig. 4b).

The total hydroxyproline content of the GC muscles in both groups remarkably increased at 4 weeks after osteotomy (non-PRP: 4.39 ± 0.08 μg/mg tissue; PRP: 4.21 ± 0.07 μg/mg tissue) and then gradually decreased at 6, 8, and 10 weeks (Fig. 4c), consistent with the findings of Masson’s trichrome staining. At 6, 8, and 10 weeks, there were no significant differences between the non-PRP and PRP groups (2.80 ± 0.06 μg/mg tissue vs 3.00 ± 0.05 μg/mg tissue, *p* = 0.45; 2.72 ± 0.17 μg/mg tissue vs 2.57 ± 0.09 μg/mg tissue, *p* = 0.33; and 2.47 ± 0.08 μg/mg tissue vs 2.41 ± 0.09 μg/mg tissue, *p* = 0.52, respectively; Fig. 4c).

## Discussion

This study, which was designed to investigate whether PRP reduced skeletal muscle fibrosis after distraction osteogenesis, found that distraction osteogenesis caused fibrous tissue formation in the GC muscles in the non-PRP and PRP groups. This fibrosis was remarkably increased at 4 weeks after osteotomy and subsequently decreased but persisted in both groups at 6, 8, and 10 weeks. Although gradual decrease of fibrosis at 6, 8, and 10 weeks reflected muscle remodeling or healing as we reported previously (Tonogai et al. 2015), we found no significant differences in total hydroxyproline content, indicative of skeletal muscle fibrosis, between the non-PRP and PRP groups at any time point.

Although distraction osteogenesis does not directly damage muscle such as attack injury, distraction osteogenesis causes severe skeletal muscle damage by pulling strength (Tonogai et al. 2015). We initially expected that PRP might significantly reduce skeletal muscle fibrosis after distraction osteogenesis. However, our study showed no positive effect on muscle following distraction osteogenesis after treatment with PRP, similar to the findings of (Delos et al. 2014). They reported a high percentage of fibrous tissue in the PRP group compared with the control group in a rat GC contusion model. We are unable to clearly explain this observation, but various factors may be involved in the process. A possible reason could be that our distraction osteogenesis model involved more severe muscle damage than simple muscle injury. Although PRP might be effective in slight or mild skeletal muscle injury, our results might suggest that severe skeletal muscle damage during osteogenesis exceeds the tissue healing function of PRP.

PRP contains various GFs. For examples, PDGF stimulates mitogenesis of mesenchymal cells and chemotaxis (Hammond et al. 2009). VEGF leads to increased angiogenesis and decreased fibrosis in injured skeletal muscle (Deasy et al. 2009; Ranzato et al. 2009). However, besides the beneficial effects, PRP also contains significantly higher concentrations of TGF-β1. TGF-β1 is a key factor in the development of fibrosis (Border and Noble 1994) because it has the ability to stimulate collagen synthesis and fibroblast proliferation contributes to increased collagen deposition (Ignotz and Massague 1986). Therefore, PRP containing TGF-β1 might potentially stimulate skeletal muscle fibrosis (Massagué et al. 1986; Allen and Boxhorn 1989; Li and Huard 2002; Borrione et al. 2010; Visser et al. 2010), although there are reports that PRP-treated muscles showed reduction of fibrosis (Gigante et al. 2012; Gigante et al. 2014; Cianforlini et al. 2015). Nevertheless, an increase in fibrosis can accelerate the fibrotic healing process after tendon and ligament injuries (Hildebrand and Frank 1998; Mishra et al. 2009; Beye et al. 2008; Fallouh et al. 2010; Huang and Whang 2010; Iqbal et al. 2011), with the presence of high concentrations of TGF-β1 in PRP potentially promoting fibrosis in injured skeletal muscle. Conversely, Li et al. reported that neutralizing TGF-β1 in PRP reduced muscle fibrosis, and Terada et al. and Huard J et al. also reported that PRP injection and administration of losartan (an antifibrotic agent) decreased muscle fibrosis in an animal muscle injury model (Huard et al. 2002; Terada et al. 2013; Li et al. 2016). Blocking of factors that cause fibrosis like TGF-β1 in PRP might be beneficial.

We selected L-PRP because it can be beneficial, stimulating the immune response against infections (Cieslik-Bielecka et al. 2007; Moojen et al. 2008); promoting chemotaxis, proliferation, and differentiation of cells; and inducing angiogenesis (Wrotniak et al. 2007). In contrast, Zhou H al. reported that L-PRP may be detrimental to the healing of injured tissue because it induces catabolic and inflammatory effects on cells and may prolong the effects in healing tissue, while pure-PRP (P-PRP) may result in formation of excessive scar tissue due to the strong potential of P-PRP to induce inordinate cellular anabolic effects when used to treat acutely injured tissue (Zhou et al. 2015). The presence or absence of WBC might also affect the results because the exact role of leukocytes in PRP is unclear as yet. Further studies are required to clarify the ideal PRP formulations including WBC.

There are limitations in this study. Of course, this result does not correspond to the clinical scenario in patients, because there is race discrepancy between human and mouse and distraction osteogeneis procedure for mouse is not exactky same as the one for human. First limitation is that muscle function, like ankle plantarflexion torque, was not measured (Tonogai et al. 2015). Analysis for TA muscles might be also necessary. Second, osteotomy site, distraction speed, period, or length might also affect the results. Third, PRP-related factors such as variations in preparations and dosage, frequency of injection, or timing of injection might affect the results. The results in this study are consistent to other authors’ findings that GFs in PRP were higher in their other studies. However, the absolute amount of the GFs was different, as Delos et al. reported PDGF-AB, VEGF, and TGF-β1 concentrations in whole blood or PRP were 125 vs 330 pg/ml, 10 vs 18 pg/ml pg/ml, and 32 vs 85 ng/ml respectively (Delos et al. 2014), Hammnod et al. reported that concentrations of PDGF in plasma or PRP was 4150 vs 20,745 pg/ml (Hammond et al. 2009), and Bir SC reported that concentrations of PDGF and VEGF in plasma or PRP was 30 vs 53 pg/ml and 958 vs 45,352 pg/ml respectively (Bir et al. 2011). The constituents of PRP might be also affected by the condition of patients or animals before blood is harvested. Additional research is needed to define the effects of these variables and applications on PRP. Finally, skeletal muscle fibrosis in this study might be affected by the fibrosis part of the bone healing/callus to some extent.

## Conclusions

In conclusion, skeletal muscle fibrosis caused by distraction osteogenesis persisted even in the GC muscles of the PRP group. At least in this study, we found no significant difference in muscle fibrosis between the PRP and non-PRP groups, and we could not show that PRP had favorable effects on skeletal muscle fibrosis. To our knowledge, this is the first report about the effect of PRP on skeletal muscle fibrosis following distraction osteogenesis. We should not use PRP for skeletal muscle following distraction osteogenesis just because PRP is a modern therapeutic approach.
